# Impact of COVID-19 history on the prevalence of coronary slow flow: a comparative study in unstable angina patients

**DOI:** 10.3389/fcvm.2026.1814606

**Published:** 2026-05-08

**Authors:** Sait Alan, Bircan Alan

**Affiliations:** 1 Department of Cardiology, Faculty of Medicine, Bolu Abant Izzet Baysal University, Bolu, Türkiye; 2 Department of Radiology, Faculty of Medicine, Bolu Abant Izzet Baysal University, Bolu, Türkiye

**Keywords:** coronary slow flow, corrected TIMI frame count, COVID-19, microvascular dysfunction, myocardial perfusion, post-acute sequelae of COVID-19, unstable angina pectoris

## Abstract

**Aim:**

This study investigated coronary slow flow (CSF) prevalence in post-COVID-19 patients presenting with unstable angina (UA) and angiographically normal coronary arteries.

**Materials and methods:**

The study included 190 patients presenting with unstable angina (UA) and confirmed normal coronary arteries via invasive angiography. Participants were divided into two age- and sex-matched groups: 95 patients with a history of COVID-19 [COVID (+) UA-NCA] and 95 patients without a prior history of the infection [COVID (–) UA-NCA]. In the study group, COVID-19 was confirmed via RT-PCR and computed tomography. All participants underwent coronary angiography to perform Thrombolysis in Myocardial Infarction (TIMI) frame count (TFC) measurements. CSF was diagnosed in patients with a corrected TFC (CTFC) ≥ 27.

**Results:**

CSF prevalence was significantly higher in the COVID (+) UA-NCA group than in the COVID (–) UA-NCA group (18.9% vs. 5.3%, *p* = 0.003). Individual CTFC values for the LAD, LCX, and RCA were also significantly higher in the COVID (+) UA-NCA group compared to the COVID (–) UA-NCA group (26.1 ± 4.3 vs. 22.4 ± 2.6 [*p* = 0.01], 24.3 ± 4.5 vs. 21.7 ± 2.4 [*p* < 0.001], and 24.3 ± 4.5 vs. 21.4 ± 2.5 [*p* < 0.001], respectively).

**Conclusions:**

CSF prevalence is significantly higher in patients with a history of COVID-19, suggesting persistent microvascular impairment.

## Introduction

Coronary slow flow (CSF) is a clinical condition characterized by anginal symptoms, angiographically normal or near-normal coronary arteries, and delayed (abnormally slow) antegrade opacification of the distal coronary vasculature (1). Although its etiology is not yet fully elucidated, contemporary evidence increasingly classifies CSF as a distinct clinical entity within the spectrum of coronary microvascular dysfunction (2). CSF is a relatively rare angiographic finding, with a reported prevalence ranging from 1% to 7% among patients undergoing diagnostic angiography for chest pain evaluation (3). Recent investigations suggest that this prevalence may be underreported, as objective diagnostic metrics like the Thrombolysis in Myocardial Infarction (TIMI) frame count are becoming more critical for identifying subtle microcirculatory resistance and ensuring the reproducibility of clinical findings (2).

The clinical presentation of patients with CSF at diagnosis is highly variable, typically including stable or unstable angina, ischemia detected on functional testing, ST-segment elevation myocardial infarction, and non-ST-segment elevation myocardial infarction. It also includes ventricular arrhythmias or sudden death, as reported in numerous case reports (4). Beyond acute events, longitudinal data now highlight that CSF is associated with an increased risk of major adverse cardiovascular events (MACE) and recurrent hospitalizations, emphasizing the clinical importance of early detection and personalized management strategies (5).

Histopathological studies have shown that CSF is characterized by endothelial dysfunction or endothelial injury in the coronary small vessels, which develops due to numerous structural abnormalities despite normal coronary arteries, including medial hypertrophy, fibromuscular hyperplasia, degeneration of endothelial cells, and endothelial swelling, leading to a reduction in luminal diameter and functional obstruction (6, 7). Recent literature emphasizes the role of chronic low-grade inflammation and oxidative stress, highlighting that various circulating inflammatory mediators and altered signaling pathways serve as key indicators of the endothelial activation that drives this flow delay (8).

Coronavirus disease (COVID-19) is characterized by an intense inflammatory response and a high incidence of thrombotic events. Autopsy studies have demonstrated severe endothelial injury associated with vascular thrombosis. In the pathophysiology of this disease, endothelial injury and dysfunction, inflammation, and thrombosis are determining factors for prognosis; therefore, COVID-19 is considered a systemic endothelial disease that leads to multiple organ failure (9). Current research has further demonstrated that SARS-CoV-2 infection can lead to persistent endotheliitis and accelerated vascular aging, which may last for several months post-infection (10).

Endothelial dysfunction plays a central role in the pathogenesis of CSF. It may occur both directly through the interaction of the SARS-CoV-2 virus with the endothelium and ACE2 and indirectly through hypoxia, hyperinflammation, and immune dysregulation, and may lead to cardiovascular diseases in patients with COVID-19 (11, 12). Emerging studies on Long-COVID syndromes have specifically linked prior infection with long-term reductions in myocardial stress perfusion, suggesting that the virus induces lasting coronary microvascular impairments that require clinical vigilance (13).

Recent studies have shown that endothelial injury can persist for 6 to 12 months post-infection due to chronic endotheliitis; therefore, we hypothesized that COVID-19 history could be associated with coronary slow flow in patients presenting with unstable angina (6–8).

Emerging clinical data during the COVID-19 pandemic suggested a potential link between prior SARS-CoV-2 infection and persistent cardiovascular symptoms, even in the absence of obstructive coronary artery disease. However, the specific prevalence of coronary slow flow (CSF) in this sub-population remains under-investigated. Therefore, the present study was designed to objectively evaluate the impact of COVID-19 history on microvascular perfusion metrics, specifically focusing on patients presenting with unstable angina and normal coronary arteries.

## Materials and methods

### Study design and population

This single-center retrospective study was approved by the ethics committee of our university (Protocol No: 2022/107) and was conducted in accordance with the principles of the Declaration of Helsinki. Hospital records were retrospectively reviewed for a period ending in October 2021. The study included patients who presented with Unstable Angina within six months following their COVID-19 diagnosis. The study group, designated as COVID (+) UA-NCA (Corona-positive Unstable Angina with Normal Coronary Arteries), included 95 patients (mean age 61.0 ± 11.6 years) with a confirmed history of COVID-19 infection. All patients in this group were hospitalized with a clinical diagnosis of Unstable Angina Pectoris, characterized by typical ischemic chest pain and objective ischemic ECG changes (such as ST-segment depression or T-wave inversion) in the absence of elevated cardiac biomarkers. All participants underwent coronary angiography due to these ischemic findings and were found to have angiographically normal coronary arteries (no stenosis >20%).

The time interval between the initial COVID-19 diagnosis (confirmed via RT-PCR and/or computed tomography) and the coronary angiography was recorded as a mean of 6.2 ± 1.4 months, representing the post-acute phase of the infection.

The control group, designated as COVID (–) UA-NCA (Corona-negative Unstable Angina with Normal Coronary Arteries), consisted of 95 age- and sex-matched patients (mean age 59.4 ± 11.4 years) without a history of COVID-19 who underwent coronary angiography during the same period for similar clinical indications (suspected unstable angina) and were also found to have normal coronary arteries.

### Inclusion and exclusion criteria

Patients with coronary artery disease that could cause endothelial dysfunction (including plaque, spasm, ectasia, or obstructive lesions defined as >20% luminal narrowing), myocardial bridging, cardiomyopathy, valvular heart disease, left ventricular systolic dysfunction (EF < 40%), or obesity (BMI ≥ 30 kg/m^2^) were excluded. Additionally, patients with chronic inflammatory diseases, active malignancy, or severe renal/hepatic failure were excluded to avoid confounding effects on endothelial function. The diagnosis of COVID-19 was confirmed in all patients by a positive SARS-CoV-2 RT-PCR test together with typical findings on computed tomography.

### Coronary angiography and TFC measurement

All angiograms were performed via the radial artery using 5F catheters with manual injection of low-osmolar contrast media. Images were acquired at a frame rate of 30 frames per second. During angiography, all patients received 100–200 mcg of nitroglycerin administered as an intracoronary injection to exclude coronary vasospasm.

Coronary angiograms were evaluated by two interventional cardiologists blinded to the clinical characteristics of the patients. In cases of disagreement, a third senior cardiologist reached a consensus. TIMI frame count (TFC) and corrected TFC (CTFC) measurements were performed. For the diagnosis of CSF, we used the TFC and CTFC methods described by Agrawal et al. (3). This method measures the number of cine frames required for contrast to reach standardized distal coronary landmarks: the distal bifurcation of the LAD (the “whale's tail”), the distal bifurcation of the segment with the longest total distance in the LCX, and the first side branch of the posterolateral artery in the RCA.

Normal TFC values were defined as 36.2 ± 2.6 for the LAD, 22.2 ± 4.1 for the LCX, and 20.4 ± 3.0 for the RCA. Due to the greater length of the LAD, the obtained frame count is divided by 1.7 to correct for its increased length, and a value of 21.1 ± 1.5 is accepted as normal. Coronary slow flow is defined as a CTFC value exceeding 2 standard deviations above the normal range for a given coronary artery. A CTFC measurement greater than 27 is considered diagnostic for CSF (3).

### Statistical methods

The descriptive data are presented as frequencies (percentages), median values [interquartile ranges (IQR)], or mean ± standard deviation. The normality of the continuous variables was assessed using Shapiro–Wilk tests and histogram graphs. For normally distributed variables, an independent samples *t*-test and one-way ANOVA were used. When the data did not meet the normality assumption, nonparametric Mann–Whitney *U*-tests and Kruskal–Wallis tests were used. Univariate and multivariate logistic regression analyses were conducted to identify the predictors of CSF, and the odds ratio (OR) for each factor was presented along with their 95% confidence intervals. The model's goodness-of-fit in the logistic regression analysis was confirmed using the Hosmer-Lemeshow test. Inter-observer variability for TFC measurements was assessed using the intraclass correlation coefficient (ICC). The analyses were performed using the Statistical Package for Social Sciences version 26.0 for Windows (SPSS Inc., Chicago, Illinois, USA). The results were considered to be statistically significant (*P* < 0.05).

## Results

A total of 190 patients were included in the study, divided into the COVID(+) UA-NCA group (*n* = 95) and the COVID(–) UA-NCA group (*n* = 95); mean ages were 61.0 ± 11.6 and 59.4 ± 11.4 years, respectively. The baseline demographic and clinical characteristics of the groups were similar, with no significant differences in age, gender distribution, or cardiovascular risk factors (*P* > 0.05) (Table 1). To ensure the reproducibility of our results, inter-observer reliability for TIMI frame count measurements was assessed; an ICC of 0.94 (95% CI: 0.91–0.97, *P* < 0.001) was found.

**Table 1:** Baseline clinical characteristics and laboratory parameters of the study population.

Parameters	COVID (+) UA-NCA (*n* = 95)	COVID (–) UA-NCA (*n* = 95)	*p*
Age	61.0 ± 11.6	59.4 ± 11.4	0.98
BMI, (kg/m^2^)	27.7 ± 1.6	26.2 ± 2.7	0.02
Sex-Male, *n* (%)	67 (70.5%)	57 (60%	0.08
DM, *n* (%)	22 (23.2%)	20 (21.1%)	0.43
HT, *n* (%)	21 (22.1%)	18 (18.9%)	0.36
Smoking	41 (43.6%)	36 (37.9)	0.25
T.Cholesterol	180.8 ± 17.9	178.5 ± 17.7	0.91
LDL-Cholesterol	127.7 ± 20.7	124.3 ± 17.1	0.050
HDL- cholesterol	51.06 ± 9.1	53.2 ± 8.1	0.45
Triglyceride	150.2 ± 19.4	145.6 ± 21	0.050
LVEF	58.7 ± 8	60.7 ± 7.3	0.68

Values presented as mean ± SD or n(%).

COVID (+) UA-NCA, Corona-positive Unstable Angina with Normal Coronary Arteries; COVID (–) UA-NCA, Corona-negative Unstable Angina with Normal Coronary Arteries; BMI, Body mass index; DM, Diabetes mellitus; HTN, Hypertension; LVEF, Left ventricular ejection fraction.

When both groups were evaluated angiographically, the prevalence of CSF was significantly higher in the COVID(+) UA-NCA group than in the COVID(–) UA-NCA group, with 18.9% (*n* = 18) and 5.3% (*n* = 5), respectively (*P* = 0.003). Specifically, the analysis of individual coronary arteries revealed that CTFC values were significantly higher in the COVID(+) UA-NCA group for all three major vessels compared with the COVID(–) UA-NCA group. The CTFC values for the LAD, LCX, and RCA were 26.1 ± 4.3 vs. 22.4 ± 2.6 (*P* = 0.01), 24.3 ± 4.5 vs. 21.7 ± 2.4 (*P* < 0.001), and 24.3 ± 4.5 vs. 21.4 ± 2.5 (*P* < 0.001), respectively (Table 2).

**Table 2:** Coronary angiography results of study populations.

Parameters	COVID (+) UA-NCA (*n* = 95)	COVID (–) UA-NCA (*n* = 95)	*p*
CSF, *n* (%)	18 (18.9%)	5 (5.3%)	0.003
LAD-CTFC	26.1 ± 4.3	22.4 ± 2.6	0.01
LCX-CTFC	24.3 ± 4.5	21.7 ± 2.4	<0.001
RCA-CTFC	24.3 ± 4.5	21.4 ± 2.5	<0.001

Values presented as mean ± SD or *n* (%).

COVID (+) UA-NCA, Corona-positive Unstable Angina with Normal Coronary Arteries; COVID (–) UA-NCA, Corona-negative Unstable Angina with Normal Coronary Arteries; CSF, coronary slow flow; CTFC, corrected TIMI frame count; LAD, Left anterior descending artery; LCX, Left circumflex; RCA, Right coronary artery.

Furthermore, a subgroup analysis was conducted by excluding all patients diagnosed with CSF from both groups [resulting in a remaining cohort of *n* = 77 in COVID(+) UA-NCA vs. *n* = 90 in COVID(–) UA-NCA] to investigate subclinical microvascular involvement. Despite the exclusion of these patients, the COVID(+) UA-NCA group continued to demonstrate significantly elevated CTFC values in the LAD, LCX, and RCA compared to the control group (Table 3).

**Table 3:** Comparison of CTFC values between COVID(+) and COVID(–) groups in the absence of overt coronary slow flow. (subgroup analysis).

Parameters	COVID (+) UA-NCA (*n* = 77)	COVID (–) UA-NCA (*n* = 90)	p
LAD-CTFC	24.3 ± 1.3	22.01 ± 2	<0.001
LCX-CTFC	22.4 ± 1.7	21.3 ± 1.8	<0.001
RCA-CTFC	22.3 ± 1.5	21 ± 1.9	<0.001

Values presented as mean ± SD or n(%).

COVID (+) UA-NCA, Corona-positive Unstable Angina with Normal Coronary Arteries; COVID (–) UA-NCA, Corona-negative Unstable Angina with Normal Coronary Arteries; LAD, Left anterior descending artery; LCX, Left circumflex; RCA, Right coronary artery.

**Figure 1:**
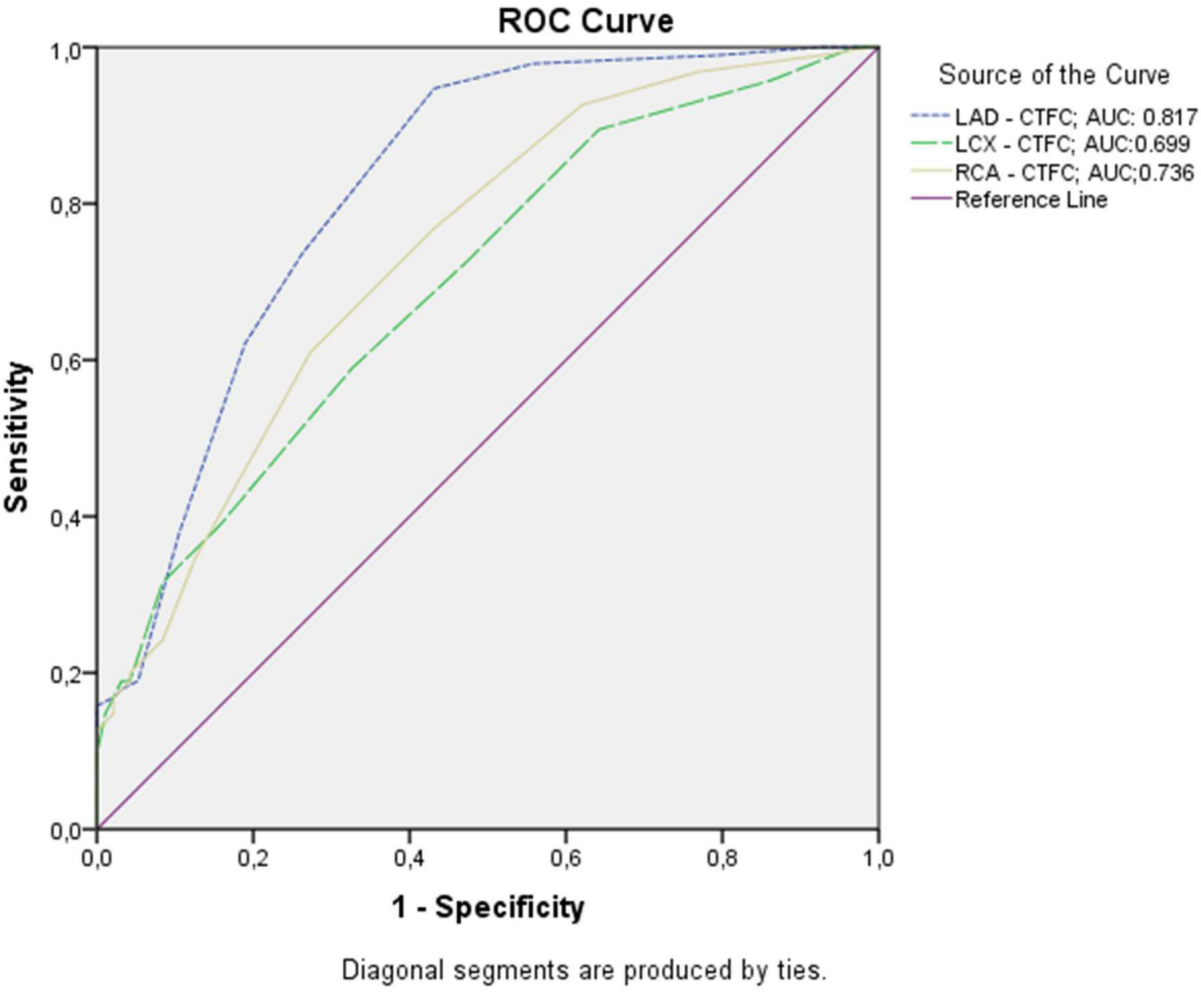
Diagnostic performance of CTFC in predicting coronary slow flow among patients presenting with unstable angina.

To identify the independent risk factors for CSF, a multivariable regression analysis was performed. Among the variables analyzed, history of COVID-19 infection was found to be the only significant independent predictor of CSF (Beta=0.214, *p* = 0.005). In this multivariate model, BMI lost its previously observed statistical significance (*p* = 0.524), and no other clinical or laboratory parameters reached significance (Table 4). Additionally, the CTFC showed high diagnostic accuracy in the ROC analysis, with significant AUC values for the LAD, LCX, and RCA of 0.817, 0.699, and 0.736, respectively (all *P* < 0.001) (Figure 1). There were no procedural complications during the coronary angiographies in either group.

**Table 4:** Multivariate linear regression analysis for predictors of coronary slow flow (CTFC).

Variable	Unstandardized Coefficient (B)	Standardized Coefficient (Beta)	*p*-value
COVID-19 History (Group)	0.140	0.214	0.005*
BMI (kg/m^2^)	0.007	0.047	0.524
LDL-Cholesterol (mg/dL)	0.003	0.182	0.107
Total Cholesterol (mg/dL)	-0.003	-0.178	0.115
Age (years)	-0.001	-0.026	0.725
Gender (Male)	0.059	0.086	0.246
Smoking (Yes)	-0.065	-0.098	0.183
Hypertension (Yes)	-0.049	-0.061	0.413
Diabetes Mellitus (Yes)	-0.011	-0.014	0.850
LVEF (%)	0.002	0.052	0.475
Triglycerides (mg/dL)	0.000	0.027	0.719
HDL-Cholesterol (mg/dL)	0.004	0.108	0.138

Dependent variable: Corrected TIMI Frame Count (CTFC).

*p < 0.01 denotes statistical significance.

BMI, Body mass index; DM, Diabetes mellitus; HTN, Hypertension; LVEF, Left ventricular ejection fraction.

## Discussion

CSF prevalence was significantly higher among the COVID (+) unstable angina patient group. Consistently, CTFC values in this group were significantly elevated compared to the COVID(–) unstable angina cohort.

In our study, CSF prevalence was found to be 18.9% for patients who presented with unstable angina and had a history of COVID-19 and 5.3% for those who did not have a history of COVID-19, with the prevalence of CSF being roughly four times higher in the COVID (+) group. Moreover, significantly elevated CTFC values may represent an important mechanism underlying ischemic symptoms in UA patients with previous COVID-19 infection. Previous studies have reported a CSF prevalence of 1%–7% in patients undergoing diagnostic angiography (1, 3). In the present study, the 5% prevalence in the COVID (–).

In a subgroup analysis performed after excluding patients with CSF from both groups, CTFC values remained higher in the COVID (+) UA-NCA group, suggesting the severity of endothelial dysfunction caused by COVID-19. This finding may indicate the need for heightened vigilance in controlling cardiovascular risk factors in patients with a history of COVID-19.

Endothelial dysfunction plays a central role in the pathogenesis of CSF (11). Endothelial dysfunction may occur both directly through the interaction of the SARS-CoV-2 virus with the endothelium and ACE2 and indirectly through hypoxia, hyperinflammation, and immune dysregulation and may lead to cardiovascular diseases in patients with COVID-19 (12). Inflammatory mediators have been shown to be associated with CSF (3, 6, 9). Endothelial dysfunction has been reported to play a key role in the pathogenesis of various COVID-19-related clinical conditions, including pulmonary thromboembolism and multiple organ infarction (14). The endothelial dysfunction associated with COVID-19 presented in current studies may explain the elevated CSF prevalence and higher CTFC values observed in the present study.

Patients with CSF are typically male, smokers, obese, and have a number of risk factors associated with metabolic syndrome (15). In the present study, risk factors for CSF, such as smoking, HT, and DM, were similar in both groups, showing that obesity is independently associated with systemic endothelial dysfunction (11). Although obese patients (BMI ≥ 30) were excluded from the study, In our study, BMI was significantly higher in the COVID(+) group, consistent with previous reports linking obesity to coronary slow flow 16. However, our multivariate analysis demonstrated that COVID-19 history is a more robust and independent predictor of CSF than BMI. This suggests that while baseline factors like BMI contribute, the primary driver of increased CSF prevalence in our cohort is the persistent endothelial dysfunction following COVID-19 infection.

Studies have reported that the most commonly affected artery in CSF is the LAD, followed, in order of frequency, by the LCX and RCA [4]. In the present study, CSF in the COVID(+) UA-NCA group affected all three arteries (LAD, LCX, and RCA).

In our study, the significantly higher prevalence of CSF at an average of 6.2 months post-infection indicates that COVID-19-related microvascular changes may persist beyond the acute phase. These findings suggest a lasting impact of the virus on coronary microcirculation, contributing to ischemic symptoms in the post-acute period (13, 17).

The main limitations of this study include its retrospective design and the lack of specific inflammatory biomarkers at the time of angiography. Additionally, COVID-19 vaccination status and the clinical severity of the initial infection were not systematically recorded, which may have influenced the degree of persistent endothelial dysfunction.

## Conclusion

The prevalence of CSF was significantly higher in individuals with a history of COVID-19. Furthermore, even in the absence of CSF, CTFC values were found to be higher in those with a history of COVID-19. Elevated CTFC values, an indicator of endothelial dysfunction, suggest that individuals who have recovered from COVID-19 may be at greater risk for cardiovascular disease.

## Data Availability

The raw data supporting the conclusions of this article will be made available by the authors, without undue reservation.
